# The ImmunoSkills Guide: Competencies for undergraduate immunology curricula

**DOI:** 10.1371/journal.pone.0313339

**Published:** 2024-11-11

**Authors:** Sumali Pandey, Samantha L. Elliott, Justine Liepkalns, Rebekah T. Taylor, Thiru Vanniasinkam, Adam J. Kleinschmit, Louis B. Justement, Archana Lal, Danielle Condry, Heather A. Bruns, Timothy Paustian, Philip F. Mixter, Rebecca L. Sparks-Thissen, Sarah Sletten, Brian D. Wisenden

**Affiliations:** 1 Biosciences Department, Minnesota State University Moorhead, Moorhead, MN, United States of America; 2 Center for Inclusive Teaching and Learning and Department of Biology, St. Mary’s College of Maryland, St. Mary’s City, MD, United States of America; 3 Department of Microbiology, Immunology, and Pathology, Colorado State University, Fort Collins, CO, United States of America; 4 Department of Biology, Frostburg State University, Frostburg, MD, United States of America; 5 School of Dentistry and Medical Sciences, Charles Sturt University, Bathurst, NSW, Australia; 6 Department of Natural and Applied Sciences, University of Dubuque, Dubuque, IA, United States of America; 7 Department of Microbiology, The University of Alabama at Birmingham, Heersink School of Medicine, Birmingham, AL, United States of America; 8 Department of Biology, Labette Community College, Parsons, KS, United States of America; 9 Department of Microbiological Sciences, North Dakota State University, Fargo, ND, United States of America; 10 Department of Bacteriology, University of Wisconsin-Madison, Madison, WI, United States of America; 11 School of Molecular Biosciences, College of Veterinary Medicine, Washington State University, Pullman, WA, United States of America; 12 Departments of Microbiology and Immunology and Pathology and Laboratory Medicine, Indiana University School of Medicine- Evansville, Evansville, IN, United States of America; 13 Department of Biomedical Sciences, School of Medicine & Health Sciences, University of North Dakota, Grand Forks, ND, United States of America; Massachusetts Institute of Technology School of Engineering, UNITED STATES OF AMERICA

## Abstract

Immune literacy garnered significant attention in recent years due to the threat posed by emerging infectious diseases. The pace of immunological discoveries and their relevance to society are substantial yet coordinated educational efforts have been rare. This motivated us to create a task force of educators to reflect on pedagogical approaches to teaching immunology and to draft, develop, and evaluate key competencies for undergraduate immunology education. The research questions addressed include: 1) Which competencies are considered important by educators? 2) Are the illustrative skills clear, accurate and well aligned with the core competencies listed in the *Vision and Change* report?; 3) What are the concerns of immunology educators about competencies and skills? We collected data on the draft competencies using surveys, focus groups, and interviews. The iterative revision phase followed the community review phase before finalizing the framework. Here, we report a hierarchical learning framework, with six core competencies, twenty illustrative skills, and companion immunology-specific example learning outcomes. Predominant themes from interviews and focus groups, which informed revisions of this framework are shared. With the growing need for immunology education across the sciences, the ImmunoSkills Guide and accompanying discussion can be used as a resource for educators, administrators and policymakers.

## Introduction

Learning frameworks are benchmarks thoughtfully developed by disciplinary experts to identify and evaluate knowledge and skills that students should develop in their course of study. Learning frameworks can be used as a guide by educators for curriculum mapping and planning using backward design principles [1]. They assist educators’ efforts to align content with classroom learning goals, and help in developing assessment tools to quantify student learning [2–4]. Competency-based learning frameworks describe skills, which are specific abilities that help students to demonstrate core competencies. These frameworks can be utilized by academic departments to assess the skills acquired throughout their programs, track progress, and provide a blueprint for future training and career opportunities in the field of immunology.

To date, several subdisciplines of the life sciences have initiated efforts to design learning frameworks [5–13]. Many of these frameworks for individual courses have been made available through the CourseSource website (https://qubeshub.org/community/groups/coursesource), and have been informed by the guiding principles published in the *Vision and Change in Undergraduate Biology Education* report [14, 15]. The *Vision and Change* report represents a consensus of biology educators, researchers and administrators focused on advancements in life science education research, life sciences research, and the future needs in STEM career pathways.

The *Vision and Change* report was published over a decade ago, and since then many tools have been developed to interpret and assess these core concepts and competencies for biology education [16, 17]. This includes the BioSkills guide that holistically considers competencies for biology programs [18, 19], or frameworks that focus on specific core competencies, for example, the process of science [20, 21], quantitative reasoning [22, 23], communication [24, 25], interdisciplinary education [26] and modeling [27, 28]. Similarly, work has been done to assess the departmental implementation of *Vision and Change* principles, and students satisfying the key undergraduate competencies [29, 30]. Many professional societies have adapted biology core concepts and competencies for their specific disciplines. For immunology education, nationwide community discussion [31–33] and application of *Vision and Change* core competencies are just beginning.

Immunology is a discipline with specific terminology, concepts and competencies [32]. Understanding how the immune system functions has direct relevance to health and disease processes that affect the global population. The COVID-19 pandemic further highlighted the importance of immunology-based research and its importance for developing vaccines and immunomodulatory approaches that are used to treat a wide spectrum of diseases. Accordingly, immunology-related curricula need significant revisions to ensure that they incorporate the call for action as outlined in the *Vision and Change* report and to ensure that those revisions use evidence-based approaches. Therefore, it became imperative to create a task force to invite the perspectives of immunology education practitioners’ and researchers’ on our draft core competencies. In 2019, we initiated a grassroots effort to invite the educator community’s perspective on concepts and competencies that are relevant to immunology education [34, 35]. The previous work by the task force examined the allocation of in-class time to different topics in immunology, and the perception of instructors concerning the relative importance of core competencies for immunology curricula [31]. Bruns et al. (2021) adopted a singular survey-based approach with over 60 respondents, which ultimately guided the immunology education task force’s discussions and led to iterative revisions of our first draft of core competencies.

Building on the work of Bruns et al. (2021), this study intended to capture the perspectives of immunology educators regarding life science competencies in the context of immunology. These perspectives informed the development and iterative revision of the competency learning framework, called the ImmunoSkills Guide, consisting of not only core competencies, but also several aligned illustrative skills and example learning outcomes. The intent was to apply *Vision and Change* core competencies to undergraduate immunology education, with actionable recommendations for curricular mapping. An approach comprising of focus groups, surveys and interviews, was used to invite community feedback to inform the ImmunoSkills Guide development [36]. The research questions that we set out to answer with this project were: 1) Which competencies are considered important by educators?; 2) Are the illustrative skills clear, accurate and well aligned with the core competency listed in the *Vision and Change* report?; 3) What are some of the common concerns shared by educators about competencies and skills in the context of undergraduate immunology education?

The task force collectively incorporated feedback iteratively to revise the ImmunoSkills Guide. During this process, the following aims were addressed:

1. Develop a hierarchical ImmunoSkills Guide that includes a set of immunology competencies and associated illustrative skills aligned with the *Vision and Change* core competencies.
2. Using focus groups and semi-structured interviews, invite and document common themes shared by immunology educators with regard to implementation of the competencies, and revise the ImmunoSkills Guide based on the feedback provided.
3. Survey disciplinary educators on the perceived importance of each immunology competency.
4. Survey disciplinary educators for the alignment of illustrative skills with the proposed immunology core competencies.

## Methods

### Participants

Immunology educators attending the 2019 American Society for Microbiology Conference for Undergraduate Educators (ASMCUE) were informally invited to discuss concept inventories and immunology curricula. Subsequent to this discussion efforts were undertaken to reach out to additional immunology educators through ASMCUE’s social media page, the American Association of Immunologists (AAI) Education Committee, and direct emails to potential participants. These efforts resulted in a fifteen-member Undergraduate Immunology Education task force, which was established in October of 2019. The members of this task force, who contributed to the project, are listed as co-authors on this manuscript, and are comprised of immunology educators, immunology researchers, biology educators and education researchers. The task force penned the first draft of the *Vision and Change-*aligned ImmunoSkills Guide. The task force also surveyed the community to gauge the relative allocation of course time to different topics in immunology, and to determine the relative importance of core competencies for immunology curricula [31].

**Fig 1:**
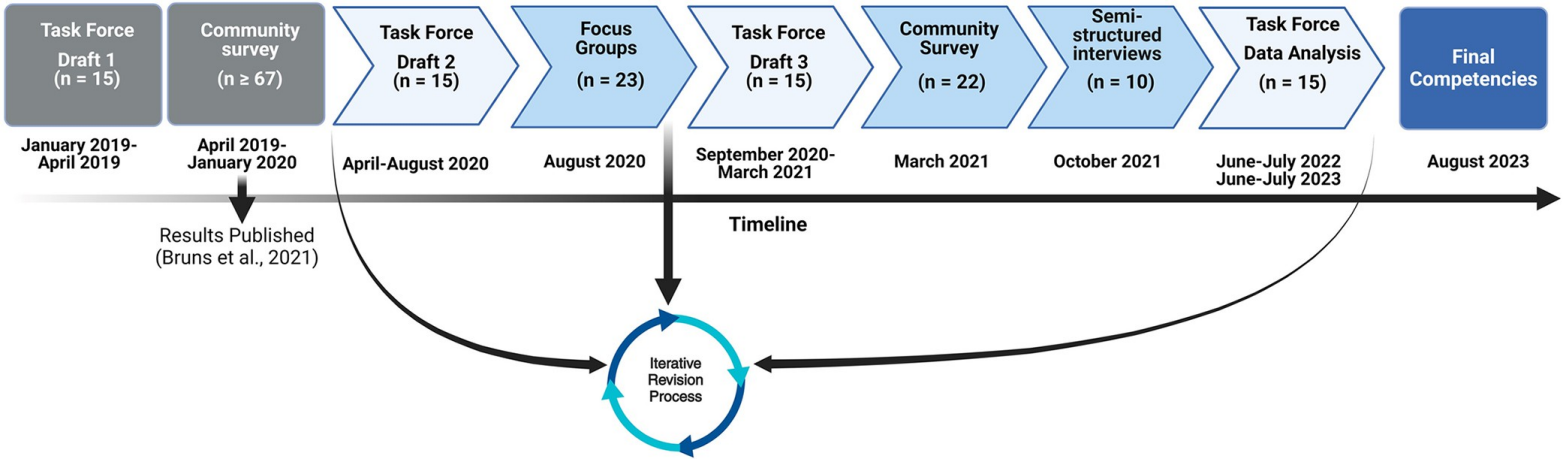
Overall process for ImmunoSkills Guide development. ‘n’ represents the number of participants in that phase of the project.

The feedback gathered during the study by Bruns et al. (2021) was collectively incorporated into the revised draft of the ImmunoSkills Guide. The second draft of the document was comprised of a three-tiered hierarchical learning framework for undergraduate immunology education. In addition to 15 individual task force members, community participants (educators) provided qualitative feedback on the ImmunoSkills Guide, through focus groups (n = 23), surveys (n = 22), and/or interviews (n = 10). Community review of the guide was followed by an iterative revision cycle by task force members (**Fig 1**). All human subject research protocols were in accordance with Minnesota State University Moorhead’s Institutional Review Board guidelines (Study ID# 1561719–3).

### Focus groups

A subset of task force members recruited and coordinated focus group participants through the Society for the Advancement of Biology Education Research (SABER) and the ASMCUE listservs. In addition, task force members sent emails to their professional contacts (**S1 File**). The call was open to all immunology and biology educators who taught immunology in varied contexts—ranging from an immunology topic in a non-immunology course (majors or non-majors) to a full immunology course or curriculum. All respondents were sent a follow-up email, which included a brief demographic survey and a link to the ImmunoSkills Guide. All focus groups were held during August of 2020 via virtual sessions. A verbal consent was obtained from each participant before proceeding with their respective session. In total, 24 participants across six sessions joined the focus groups. The guiding questions and the timing of each question was coordinated and shared online documents were distributed to focus group moderators to ensure consistency. The virtual meetings were organized such that each virtual focus group included at least 1–2 experts in immunology and included both teaching-focused and research-focused faculty or staff. Task force members facilitated breakout sessions using a guiding script (**S1 File**) with 2–4 participants in each session, and each focus group had their own virtual breakout space with a shared screen of the list of select competencies and skills they were to address. Participants shared their responses orally and were free to use the chat function. The data collected from the virtual focus group session was shared amongst the facilitators during a debrief session, immediately following each focus group session. The sessions were recorded and stored on a password-protected Google Drive shared amongst the co-investigators of this study. Thereafter, task force members iteratively revised the guide in consultation with the entire task force. The revised guide was then assessed by a survey, which was done alongside the interviews.

### Surveys

The survey was designed by S.P. using Qualtrics^XM^ survey software (Seattle, WA, U.S.A.), and piloted by the task force members before dissemination via professional society listservs (SABER, ASMCUE and the Society for Leukocyte Biology (SLB)), e-mails to professional contacts of the task force members, and social media. The survey consisted of the following sections: 1) a written and video primer to describe the hierarchical ImmunoSkills Guide used for organizing the core competencies, illustrative skills and example learning objectives; 2) demographic information related to the participant’s teaching area/expertise and institutional affiliation, and 3) five-point Likert scale-based questions to evaluate the importance of each core competency for undergraduate immunology education, alignment of skills with each core competency, and an open text box for participants to note their comments with regards to core competencies and illustrative skills (**S2 File**).

### Interviews

Reviewing the ImmunoSkills draft in its entirety is a time-consuming process for an individual. In focus groups and surveys, the respondents had an option to comment on one or more competencies based on their availability. With the interviews, we wanted the reviewer to go through the entire draft so that they could ensure the depth and breadth of coverage. Therefore, e-mail addresses were gathered from focus groups and survey participants. While the survey responses were gathered anonymously, the participants had the option to note their e-mail IDs if they wanted to be approached for reviewing the subsequent drafts of the Guide. From the participants who shared their e-mail IDs, twenty participants were randomly invited to review the ImmunoSkills Guide draft and participate in an interview. Of these, nine faculty consented to the interview. An additional immunology educator was invited to participate due to their specific expertise in Ecoimmunology- an expertise not represented in the focus groups. Focus group participants also noted that immunology should be more inclusive of non-human work, which we wanted to address with the Ecoimmunology expert. The interviews were conducted using a semi-structured approach [37] and verbal consent was obtained from each participant at the beginning. The revised draft of the ImmunoSkills Guide (post-focus groups), the Qualtrics^XM^ survey, and the interview prompts (**S3 File**) were shared with interviewees at the time of scheduling the interviews. The interviewer shared a screen and narrated each core competency and illustrative skill to the participant, followed by a prompt to provide feedback on the competencies and illustrative skills. Interviewees either agreed to the core competencies and illustrative skills as listed, or paused and expressed their concern, commented, or questioned before moving on. Afterwards, each interviewee was compensated for their time with a $50 Amazon gift card.

### Qualitative data analysis

We adhered to previously reported guidelines for qualitative data analysis [38]. All focus group session recordings were transcribed using Otter.ai (Mountain View, CA, USA) to ensure feedback was captured accurately and in its entirety. Recordings were annotated to indicate where the participants discussed, paused, or commented on a competency or illustrative skill. Thereafter, a subset of task force members served as coders for recordings related to each core competency. Three of these coders were not involved in the data collection through focus groups and only one of the coders was involved in data collection through interviews. We adopted an inductive approach to codebook development [38]. This included individual coding followed by team discussion and revision of the codebook until no new codes emerged and all coders approved. The final codebook consisted of eight codes that captured key themes from the data (**S1 Table**). Coders reviewed the annotated recordings and used the final codebook to assign codes to each participant’s comment on a core competency and/or illustrative skill. Throughout the coding process, the goal was to reach a consensus, which allowed each coder to discuss nuances and patterns in the data with the other coders. At least three coders reached a consensus on each of the assigned codes.

### Statistical analyses

This study provides descriptive statistics (relative frequencies) for various assessment measures. The data were analyzed and graphed using Microsoft Excel and Graph Pad Prism 10.0, respectively.

## Results

### The ImmunoSkills Guide developmental process

In the previous study, competencies were reviewed by the educators (n = 67–68) where respondents rated the relative importance of core competencies for immunology curricula [31]. The overall feedback noted at the time was that educators wanted to see additional specific examples related to the competencies. Thereafter, the task force drafted the second draft, and this process was guided by the literature [31, 39–42] and task force members’ experiences with their own classrooms. The fifteen-member task force first wrote a comprehensive list of learning outcomes. We also invited learning outcomes through community events such as posters, workshops, and interactive sessions at conferences (ASMCUE, AAI, SABER and SLB). We then distilled these learning outcomes to illustrative skills nested under the core competency. We arrived at the list of illustrative skills when all the learning outcomes we had, could be nested under one of the listed illustrative skills. The twenty illustrative skills nested under the core competencies are non-redundant.

Accordingly, the second draft of the ImmunoSkills Guide is structured in a three-tiered, hierarchical format with three levels ordered by their relative importance for undergraduate education (**Table 1**). The foundational level of this guide includes core competencies adopted and/or modified from the AAAS *Vision and Change* report for undergraduate biology education [14]. This top level of core competencies is considered critical for undergraduate immunology students to develop. The next level features illustrative skills aligned with each core competency, which are described as specific abilities that can help students demonstrate these core competencies and allow students to delve deeper into the immunology curriculum. Finally, immunology-specific example learning outcomes based on the illustrative skills and core competencies comprise the third level, which is listed to suggest ways that educators may deploy this guide within their classroom or program.

**Table 1:** The ImmunoSkills Guide.

Core Competency	Illustrative Skill At the end of an immunology course/module/topic, students will be able to:	Example Learning Outcomes
1. Ability to apply the process of science	1.1. Locate and evaluate peer-reviewed articles pertaining to immunology	• Compare the information in an immunology-related news article with the original scientific source • Compare the relative level of scientific evidence provided by: a primary research article, a systematic review, and a meta-analysis on an immunological topic
	1.2. Critically analyze key findings and experimental design within primary immunology literature	• Identify the most critical data that supports the hypothesis in an immunology research article • Identify the sources of implicit bias within experimental designs in immunological literature
	1.3. Design an experiment to address an immunology-based research problem	• Write a mock research grant proposal to address an immunological question
2. Ability to understand the relationship between science and society.	2.1. Identify inaccuracies in popular media about immunological topics that are consumed and shared by the lay public	• Determine the scientific accuracy of a social media post (e.g., a meme, a blog article, a video) about the immune response against a pandemic virus.
	2.2. Discuss the impact of immunological research on society	• Describe how cancer immunotherapies have impacted public perceptions of the disease • Design an advocacy poster to address the societal and ethical implications of vaccination programs • Discuss the sources of implicit bias that may impact immunological research
3. Ability to communicate and collaborate with others	3.1. Present an immunological topic at a level appropriate to the intended audience	• Create and present a poster about recent immunological findings to peers or disciplinary experts • Write a blog article about recent immunological findings for the general public
	3.2. Contribute within a team to move a task forward	• Assemble an interdisciplinary team, and collaborate to write a white paper that addresses an immunology related real-world problem
	3.3. Contribute within a team to promote a positive environment	• Describe your approach to gather and give feedback, and achieve consensus within your team • Reflect on and articulate your own and your teammates’ contribution to an immunology-related project
	3.4. Demonstrate an ability to manage conflict	• Use active-listening techniques within a role-play situation to mediate a health-related conflict.
4. Ability to use quantitative reasoning	4.1 Apply statistics to analyze immunological data	• Choose an appropriate statistical test to analyze an immunological data set and justify your choice • Describe the assumptions and potential biases of the chosen statistical tests.
	4.2 Interpret different types of graphical representation of immunological data	• Interpret a flow cytometry histogram
	4.3 Draw meaningful conclusions from an immunology-related data set	• Compare your interpretation of the data in a figure from an immunological research paper with conclusions drawn by the author
5. Ability to perform basic lab procedures	5.1 Use standardized safety practices in an immunological laboratory	• Demonstrate competence in federal and institutional regulatory protocols (e.g. Biosafety levels, IACUC, OSHA, IRB)
	5.2 Use best technical practices in an immunological laboratory	• Demonstrate competence in using basic lab equipment (e.g. pipettes, autoclave and centrifuges) and methodologies (e.g. aseptic technique, use of appropriate Personal Protective Equipment, dilution, lab math, animal handling, general chemistry skills, basic microscopy)
	5.3 Use best record-keeping practices in an immunological laboratory	• Document and report on experimental protocols, results and conclusions in a lab notebook
6. Ability to explain and/or perform laboratory methodology to address an immunology-based research question	6.1 Identify and/or isolate immune cells	• Identify specific leukocyte populations on a fixed blood smear slide • Explain how to isolate naive T lymphocytes from a mouse spleen • Explain how monoclonal antibodies can be used as antigen detection tools in cells and tissues • Score the extent of inflammation in histological tissues obtained from a diagnostic lab
	6.2 Measure effector functions of immune components	• Measure phagocytic uptake by immune cells using a fluorescence-based assay • Explain how antigen-specific lymphocytes can be tracked in an organism using flow cytometry or intravital microscopy
	6.3 Detect the presence of an antigen or an antibody	Use an immunoassay to detect the presence of a cytokine in a cell culture supernatant • Use a hemagglutination assay to determine blood types
	6.4 Measure the immune response upon manipulation of an experimental system	• Compare the immune response of a wild type and a genetically modified organism against a pathogen • Predict/assess the effect of a drug on the immune response of *Arabidopsis* to a pathogen
	6.5 Use modeling/simulation for an immunology-based investigation	• Use a computer-based model for HIV infection to determine the lifespan of infected cells • Compare and contrast immune system organization in a model organism (e.g., *Caenorhabditis elegans*, zebrafish, *Drosophila*, *Arabidopsis*) to humans • Using role-play, simulate cytokine-mediated immune response to a pathogen of your choice

The document was revised and reviewed using focus groups, surveys and interviews. Given the first-of-its-kind approach to align immunology-specific core competencies with the *Vision and Change* report, this project involved informing the educators through videos and explanatory preambles, about the project, the overarching themes emerging from learning outcomes and the relevance of this approach to pedagogy, before requesting them to share their feedback. We constantly looked for missing content from our framework. The importance of the core competencies, as well as the alignment, accuracy, and clarity of each illustrative skill, was reviewed by the study participants. Although the example learning outcomes were available to the participants, we did not ask them to specifically review those outcomes. By making these learning frameworks available on the Course Source website [13], we intend to invite educators to write their learning outcomes and nest them under a given core competency or an illustrative skill. We believe that most of the specific learning outcomes that educators propose can be nested under one of the illustrative skills and core competencies. The framework will help educators adopt backward design, focus on core competencies and overarching illustrative skills for the assessment and instructional purposes.

### Demographics

**Fig 2:**
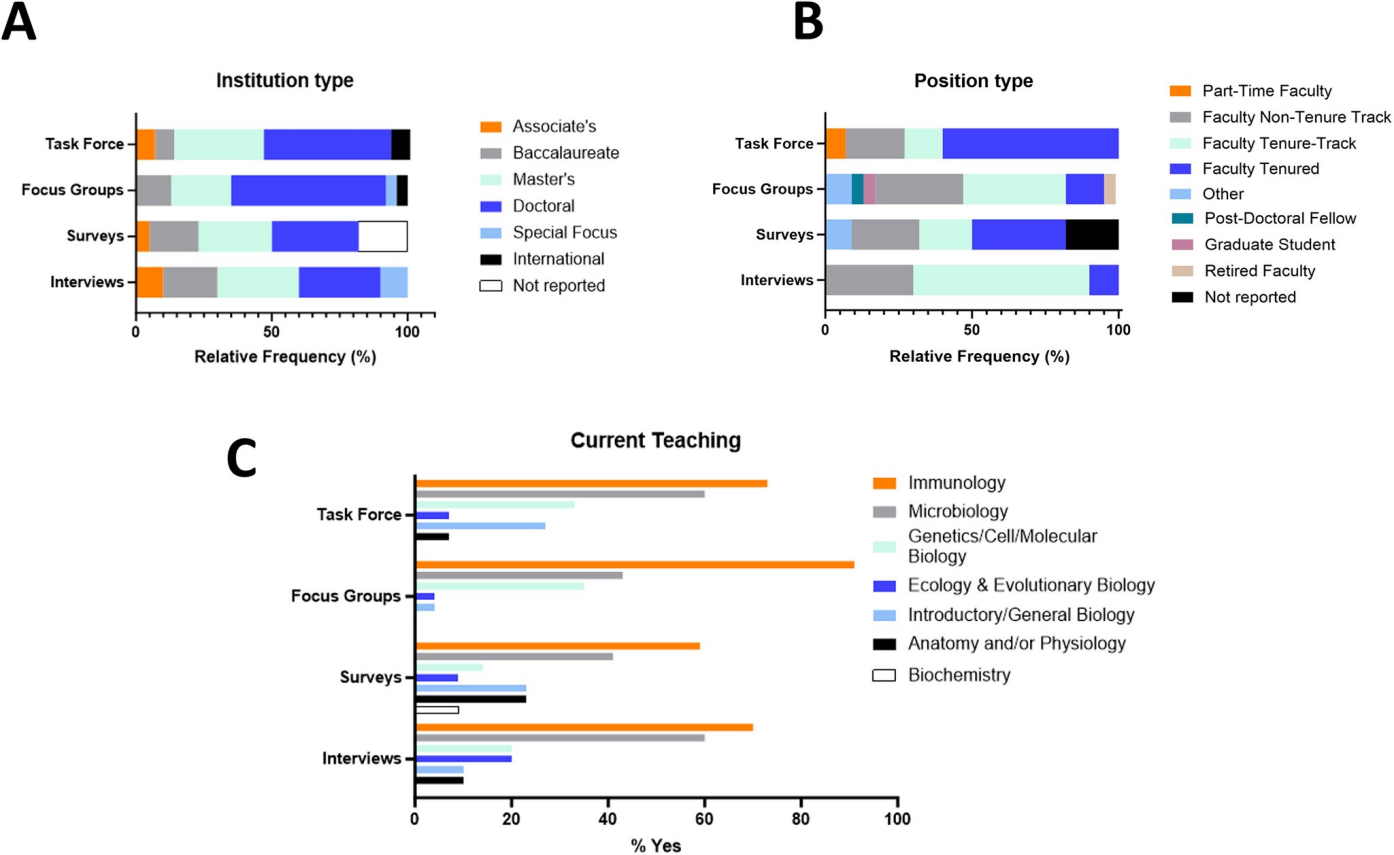
Demographics. The institution type (A) and professional position type (B) for each member of the task force, focus group, survey or interview participant pool is depicted. In addition, the stand-alone courses being taught by these participants are depicted in (C). Most of the undergraduate faculty taught more than one course.

Nearly 40 immunology education community members from a wide range of institution types and geographic locations provided qualitative feedback on the ImmunoSkills Guide, through task force contributions (n = 15), focus groups (n = 23), surveys (n = 22), and/or interviews (n = 10) (**Fig 2**). A diverse range of institution types were represented with most (greater than 50%) of the institutions being doctoral or master’s granting based on the Carnegie classification of institutions (**Fig 2A**). Several types of positions were represented among respondents, including academic faculty, graduate students, post-doctoral fellows, and lab scientists. Greater than fifty percent of respondents were either tenured or tenure-track faculty (**Fig 2B**). Immunology was the subject that most (greater than 55%) of the respondents were teaching, and several faculty taught a variety of biology and/or biochemistry courses in addition to immunology (**Fig 2C**).

### Common themes identified by focus groups

**Fig 3:**
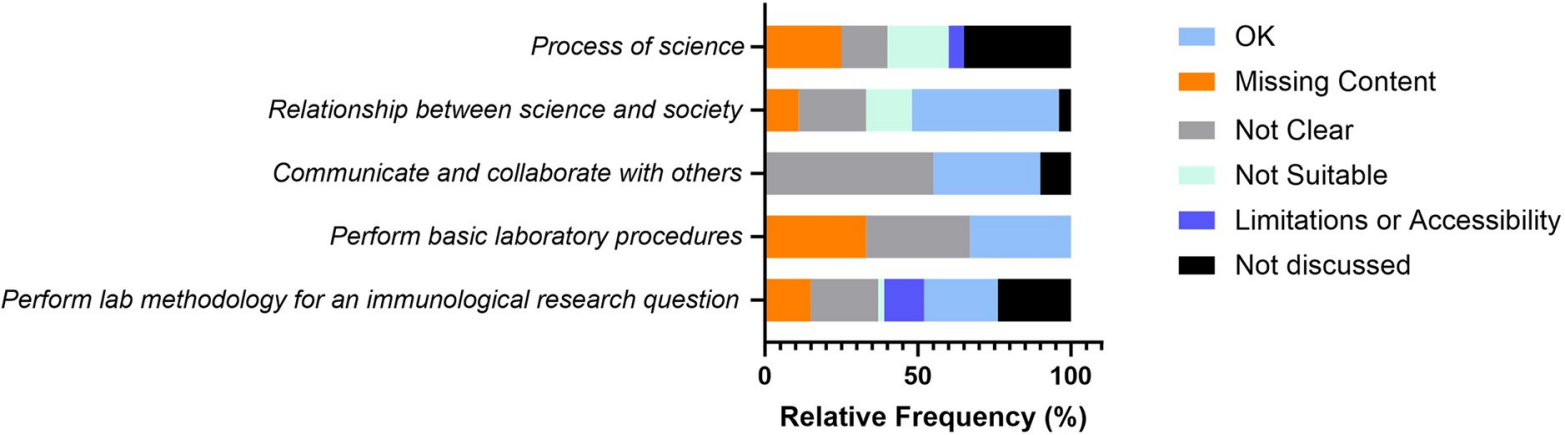
Relative frequency (%) of the concerns or consent in the qualitative data obtained from focus groups participants. Focus group participants reviewed draft 2 of the ImmunoSkills Guide. OK indicates consent by the focus group participant, where they identified no issue with the core competency or the illustrative skill. Concerns raised by the participants in the core competency or illustrative skill were classified as those associated with missing content, lack of clarity or vague word choice, lack of suitability for their course level or structure, or limitations with covering that particular competency or skill in their courses. If the competency or skill was not discussed by the participant, then we classified it as ‘not discussed’.

Focus group participants reviewed draft 2, debated over several word choices, and discussed concerns with the competencies and the illustrative skills. As depicted by the light blue bars in **Fig 3**, the percentage of individuals in the focus groups who agreed with the competencies was less than fifty percent. The themes from the focus group discussions included concerns with missing content, clarity, suitability for their course, or limitations with access to resources or time for an immunology laboratory (**S1 Table**), and were supported by example quotes for each theme that emerged (**S2 Table**). This aggregated feedback informed the ImmunoSkills Guide draft revision process, resulting in a third draft.

### Competencies perceived as important by immunology educators

**Fig 4:**
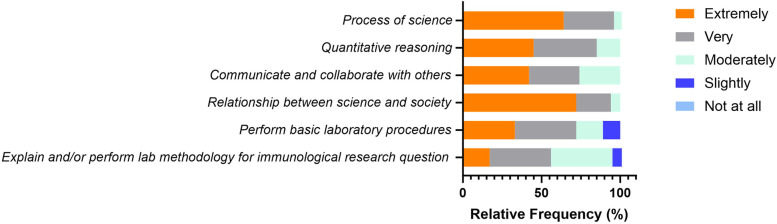
Perceived importance of each competency, as rated by the survey participants. For each core competency, participants responded to the question “How important is this competency for an immunology student to develop?” (5 = Extremely important and 1 = Not at all important). The % of respondents (Relative Frequency) corresponding to each choice are represented.

The third draft was then reviewed by survey participants, where respondents were requested to rate the importance of core competencies in the context of undergraduate immunology education. Greater than 50% of survey participants rated all five competencies as extremely or very important, and none of the participants considered any of the competencies as “Not at all important” (**Fig 4**). While none of the competencies was rated as “not at all important”, two competencies “Perform Basic Laboratory Procedures” and “Explain and/or Perform lab methodology for immunological research question”, were perceived as slightly important by 11 and 6% of participants, respectively.

### Relative alignment of illustrative skills

**Fig 5:**
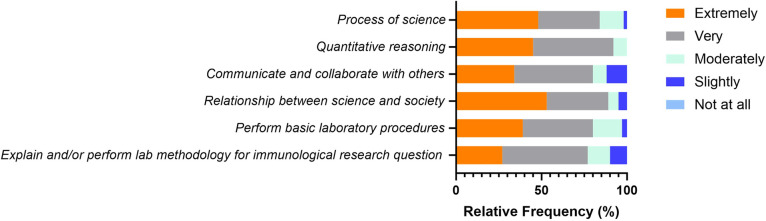
Relative alignment scores, as rated by the survey participants. For each illustrative skill noted under the core competency, participants responded to a Likert-based question “In your opinion, how well does the given illustrative skill align with the core competency?” (5 = Extremely Well Aligned and 1 = Not well at all). The % of respondents (Relative Frequency) corresponding to each choice are represented.

The third draft was also reviewed by survey participants for relative alignment of each illustrative skill to the core competency under which it was nested). Greater than 77% of survey participants rated the illustrative skills as extremely or very well aligned with the core competencies, and none of the participants considered any of the illustrative skills as “Not at all well aligned” (**Fig 5**).

### Common themes from the semi-structured interviews

**Fig 6:**
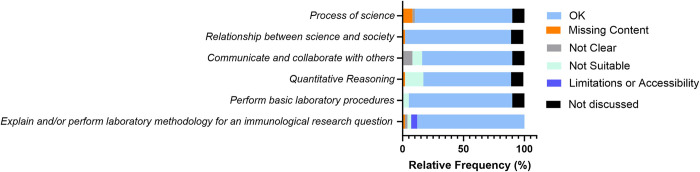
Relative frequency (%) of the concerns or consent in the qualitative data obtained from interviewees. Interviewees reviewed draft 3 of the ImmunoSkills Guide. OK indicates consent by the interviewee, where they identified no issue with the core competency or the illustrative skill. Concerns raised by the interviewees in the core competency or illustrative skill were classified as those associated with missing content, lack of clarity or vague word choice, lack of suitability for their course level or structure, or limitations with covering that particular competency or skill in their courses. If the competency or skill was not discussed by the interviewee, then we classified it as ‘not discussed’.

The interviewees reviewed the third draft of the ImmunoSkills Guide. The iterative development of the ImmunoSkills Guide based on community feedback resulted in a higher percentage consent relative to previous versions, as depicted by light blue bars in **Fig 6**. Minor concerns included missing content, clarity, importance, suitability for their course, and limitations associated with covering that competency/skill. These themes are explained (**S1 Table**), and example quotes for each theme emerging from the interview data are noted in (**S3 Table)**. The thematic analysis of the feedback obtained through interviews was analyzed by the task force, which then helped us to draft the final ImmunoSkills Guide (**Table 1**). Each point raised by the interviewees was discussed by the task force members and a decision was made on the best course of action with regards to changes to the ImmunoSkills Guide,. Based on the collective feedback obtained through this study, the following suggestions were incorporated in the final version of the ImmunoSkills Guide:

1. An interviewee identified the need to include phrases that would help identify the sources of implicit bias and structural racism associated with scientific research. Upon discussion, the task force addressed this feedback with an example learning outcome, noted under competencies #1, 2 and 4.
2. The words “primary and secondary literature” were not immediately clear to one of the interviewees when discussing competency #1. Therefore, task force members decided to add the word “evaluate” to illustrative skill #1.1 (Locate and *evaluate* peer-reviewed articles pertaining to immunology) and omit the illustrative skill #1.4 (Distinguish between primary and secondary immunology literature), to avoid redundancy.
3. With regards to competency #3, the task force intentionally used a broad term “others” to allow educators to tailor “others” to their specific classroom needs. This intent was not entirely clear to one of the interviewees, who recommended adding both; scientific and public audience, with regards to presenting an immunological topic. We addressed this by adding clarifying words “at a level appropriate to the intended audience” to illustrative skill #3.1 (Present an immunological topic at a level appropriate to the intended audience). We also modified the example learning outcomes associated with this illustrative skill, to indicate different types of audience—general public, peers and disciplinary experts. Under this same competency, we noted a few soft skills related to illustrative skills.
4. Task force members debated over the words “best” versus “standardized” versus “appropriate” for illustrative skills #5.1–5.3. The word standardized was used for safety practices, since there are federal guidelines available for these through IACUC, OSHA and IRB. For technical and record-keeping practices, we decided on the word “best” such that instructors have a choice to define best for their class, based on the task-appropriate practices that are considered best at the time in the community and based on their student learning outcomes.
5. For core competency #6, the word “perform” was replaced with “explain and/or perform” to emphasize the importance of “explain” as a core competency for undergraduates, and to clarify that instructors have a choice between having their students explain the laboratory methodologies or perform them, or do both.
6. An interviewee emphasized the importance of histology for undergraduate education due to its relevance for medical lab science and pathology assistant careers. Therefore, we included histology based example learning outcomes under competency #6.
7. Lastly, since access to immunology laboratory reagents and research models can be a limitation at many institutions, we included example learning outcomes that use non-murine model organisms (e.g., fish, invertebrates and plants) or role-play activities that do not require computers or proprietary software.

## Discussion

Here, we present an ImmunoSkills Guide that outlines core competencies for teaching immunology at the undergraduate level. This ImmunoSkills Guide was generated by multiple rounds of revision based on feedback from educators who teach immunology in diverse contexts. The final ImmunoSkills Guide features six core competencies and twenty illustrative skills (**Table 1**). By adopting several different approaches to data collection, including focus groups, surveys and interviews, the task force was able to thoroughly evaluate wording and intentions in crafting the guide, such that it aptly represents the educator’s perspectives for implementing an immunology competency-based curriculum in the classroom. As a result, the ImmunoSkills Guide can be confidently adopted by instructional designers, curriculum planners and administrators for course and/or program design, assessment, and for faculty evaluation.

It is our intention that this guide be used as a roadmap rather than a mandate, and that it encourages instructors to adopt a competency-based approach for immunology education. It is not our expectation that instructors will necessarily cover all six competencies and illustrative skills in one course. Instead, depending on the course and programmatic context, educators may focus on a subset of these competencies within an individual course or address the entire set of competencies across multiple courses in the undergraduate curriculum.

Immunology is taught in varied contexts within the undergraduate curriculum. Dedicated immunology majors are not widely represented in the U.S. higher education scene, with only one such reported major at the University of Alabama at Birmingham (https://nces.ed.gov/ipeds and [43]. Often immunology is taught as a graduate level course, an upper-level elective within the undergraduate curriculum, or within the context of microbiology or human anatomy and physiology. At community colleges, immunology is either not covered at all, or it is covered as a component of microbiology or human anatomy and physiology; but it is not taught as a stand-alone course, with the exception of medical lab science programs. Immunology may also be covered in some non-major courses, such as introduction to Public Health and vaccines [32]. The landscape in the U.S. is also not representative of what happens globally [44]. For example, in the United Kingdom, secondary-school biology students are expected to learn about body’s defence mechanisms against pathogens [45].

In the absence of a full immunology major, parts of the ImmunoSkills Guide may be adopted based on individual needs of an instructor, or the students. For example, whereas a non-majors class may focus entirely on locating and evaluating immunology-based news articles [46], an upper-level class may review primary immunology literature [47, 48] or write immunology-focused mock grant proposals [49]. Similarly, introductory biology students may focus on basic graphical interpretation [50], whereas advanced undergraduate students may engage in activities focused on learning immunological techniques, collecting and analyzing data to conduct scientific inquiry or writing a journal-style manuscript [40, 51]. Mapping these activities against the ImmunoSkills Guide may then help educators and students assess the depth and breadth of coverage of immunology-related skills within their curriculum and plan interventions and assessments to meet specific learning outcomes.

The ImmunoSkills Guide presented here represents three differences from the *Vision and Change* Core Competencies. Firstly, the two core competencies listed in the *Vision and Change* report “Communicate and Collaborate with other disciplines” and “Ability to tap into the interdisciplinary nature of science” were replaced with one single competency, i.e. “Communicate and Collaborate with Others”. The decision to combine this competency into one was made within the task force when the task force attempted to write the learning outcomes for these competencies. To meet the competency, “Ability to tap into the interdisciplinary nature of science”, communicating and collaborating with other disciplines is an important step. Therefore, instead of having a stand-alone competency—“Ability to tap into interdisciplinary nature of science” we have a learning outcome—“Assemble an interdisciplinary team and collaborate to write a white paper that addresses an immunology related real-world problem”. This learning outcome was nested under the competency—“Ability to communicate and collaborate with others”, where ‘others’ could include experts from other disciplines. Secondly, similar to Microbiology Curriculum Guidelines (Merkel and Microbiology, 2012), we included two lab-based competencies; Competency#5: Ability to perform basic lab procedures, and Competency#6: Ability to explain and/or perform laboratory methodology to address an immunology-based research question. Technical skills are hugely important to progress in immunology discipline. While competency#5 uses ‘perform’ as an action verb, competency#6 states explain and/or perform as suggested by our community reviewers and discussed in the next paragraph. By separating these two competencies, we could clearly emphasize the relevant action verbs and the importance of each competency in context of undergraduate immunology education. Thirdly, instead of listing “Modeling and Simulation” as a core competency, we nested it under competency#6 (illustrative skill #6.5), because while critical for immunologists, modeling and simulation are not the only but one of the several means to address an immunology-based research question.

Immunology-focused laboratory instruction offers significant benefits for undergraduates, including exposure to cutting-edge techniques and a comprehensive, interdisciplinary curriculum. These labs can help prepare students for pathology assistant jobs, medical lab science programs, histo-technician jobs, graduate and professional schools [39, 52–55]. Yet, access to an immunology lab was noted as a significant limitation by educators in our previous work [31] and in this study (**S2 and S3 Tables**). This concern stemmed from limitations of curriculum design, faculty workload, and the need for cost-prohibitive resources (e.g., flow cytometry, reagents, or limited access to research models such as mice). As a solution to this limitation, community feedback helped the task force clearly emphasize the importance of “explaining immunological methodologies” versus “performing immunological techniques” for undergraduate students. Educators may use the American Association of Immunologists Recommendations (Table III in [33]) to design specific learning outcomes that can be nested under core competency and illustrative skills noted in this guide. The final guide notes several example learning outcomes for competency #6, which may be accomplished with virtual labs (e.g., HHMI’s virtual ELISA, bioinformatics for antibody) or simulation-based software (e.g., cellcollective.org or Labster) or could be modeled within a classroom using readily available supplies (e.g., Legos for VDJ recombination, bouncy balls for flow cytometry) or role-play based activities [56–59].

Competency #5 focuses on basic laboratory training, including safety guidelines, using basic lab equipment properly and recordkeeping. Basic laboratory training is recognized as a core competency due to its importance for training undergraduate immunology students for future careers, and it is acknowledged that this does not necessarily require an immunology lab, but could be covered in other courses in the biology curriculum (e.g., cell biology, genetics, microbiology or biochemistry labs). Also, immunology-specific example learning outcomes for all illustrative skills noted under competencies #1–4 can be executed in a lecture or a lab setting. This recommendation is based on evidence for the effectiveness of curriculum aligned with *Vision and Change* core competencies to improve scientific literacy, where improvements could be achieved through active learning exercises centred on scientific literature and not necessarily through increasing laboratory exercises [60–62].

Upon surveying the educators regarding the perceived importance of core competencies for undergraduate immunology education, it was observed that the ability to apply the process of science was ranked highest in importance, followed by both the ability to communicate and collaborate with other disciplines and the ability to understand the relationship between science and society. The ability to use quantitative reasoning was ranked next, with the ability to use modeling and simulation in immunology ranked the lowest in importance [31]. In the current study, the ability to understand the relationship between science and society trended to the top in terms of its importance. This could be partially explained by the timing of this study, where the community was just coming out of the COVID-19 pandemic, and evidence for vaccine hesitancy, misinformation, and a general lack of immune literacy was prevalent [63, 64]. In addition, COVID-19 forced an online pivot for educators, which led to further evaluation of existing curricula from an online teaching perspective [65].

### Limitations and future directions

Our intent behind employing multiple methodologies in this study was to capture the nuances of educator feedback while reviewing this ImmunoSkills Guide. At the end of the iterative revision process, we received strong overall support for the ImmunoSkills Guide, and minor suggestions made during the interviews were addressed via discussions within the 15-member task force.

Immunology represents a niche discipline in most educational settings within the United States. This was evident in a lower participation rate in our study, as compared to similar projects in other fields [19]. This modest participant pool, as compared to the broader biology education community, is evident in previous such community-based attempts for undergraduate immunology education (e.g. [31, 33]. The number of respondents who reviewed the drafts represents a fraction of immunology educators globally. While all types of institutions were represented in our data, we acknowledge that the list is not comprehensive. The iterative process and the qualitative approach used in the study allowed us to gather in-depth feedback to critically evaluate the ImmunoSkills Guide.

This study gathered feedback from educators and thus lacked the perspective of professionals in the pharmaceutical and biotechnology industries. Many authors and reviewers of the competencies have recent or ongoing ties to applied research, therefore we expect that the competencies highlighted here are representative of foundational skills that undergraduates need to be exposed to. However, real-time data from the labor market, if available, could be very informative in improving the curriculum. Future work also entails designing learning resources and assessment tools (e.g., grading rubrics and question banks) specific to each competency and skill in the guide. As noted by one of our interviewees and previously by Riestra et al. [66] our task force recognizes that addressing implicit bias within the context of immunology competencies is important, and although it wasn’t included as a core competency in this guide, we will continue to identify resources and pedagogical tools to address diversity, equity and inclusion within the immunology curriculum.

## Conclusions

The higher education landscape continuously evolves and is influenced by many factors including the changing demographics of our society, the need for varied modalities (e.g., face-to-face, virtual) to meet situational demands, and a constant tug-of-war between resources and enrollments. This emphasizes the importance of periodically reevaluating curricula. The role of immunology in managing public health is more important than ever and the preparation of undergraduate students is not complete without a solid foundation in clearly defined core competencies and illustrative skills. The ImmunoSkills Guide developed by this task force with community input includes recommendations and perspectives of teaching faculty, and is a ready-to-use resource for educators to use. This guide can serve as a template for curriculum development and assessment for programs that do not yet fully address immunological topics and help expand and validate those that do. We hope that this first-of-its-kind document in the immunology education space will invite and encourage educators to develop, teach and assess undergraduate competencies and skills in the context of immunology.

## Supporting information

S1 File Appendix 1—Invitation template used to recruit focus group participants. (DOCX)

S2 File Appendix 2—Survey instrument. (DOCX)

S3 File Appendix 3—Interview script. (DOCX)

S1 Table The iteratively developed codebook used to analyze the qualitative data. (DOCX)

S2 Table Thematic analysis of focus group discussion. (DOCX)

S3 Table Thematic analysis of interview data. (DOCX)
