# Supplementary material for: The ImmunoSkills Guide: Competencies for undergraduate immunology curricula
Source: PLoS One. 2024 Nov 11;19(11):e0313339. doi: 10.1371/journal.pone.0313339 (PMC11554037; doi:10.1371/journal.pone.0313339)
Supplement: S1 File — (DOCX) [file pone.0313339.s001.docx]

**Supporting Information**

**S1 File. Appendix 1** **- Invitation template used to recruit focus group participants**

Dear ______,

Would you be interested in participating in the review process of a set of undergraduate immunology curriculum guidelines generated by a task force of colleagues? We would like feedback from a range of immunology educators: from teaching an immunology module within a biology course (for majors or non-majors) to teaching a full undergraduate immunology course.

Please consider registering for our focus group by filling the form below by August 5th:

Link to a short Google Form

We would love your feedback in making this set of curricular guidelines broadly applicable!

**Focus Groups**

After joining the main Zoom room, participants were organized into groups of 3-4 per breakout session, with one-two facilitators per session. Each breakout session was scheduled for a total of 40 minutes with 10-15 minutes per competency and set of illustrative skills (IS). Verbal consent, information on courses taught and institution name was gathered from each participant at the beginning of the session. During the session, the competency and the illustrative skills were projected through screen sharing, and the following questions were asked for each competency:

1. **Questions related to the competency:**

- Explain to each other the above competencies.
- Is this core competency clear? Too vague?
- Is the core competency accurate? Important for undergraduate majors?

1. **Questions related to illustrative skills (IS):**

- Explain to each other what the illustrative skills mean and how each is significant for undergraduate majors
- Do you see any issues with clarity/vagueness?
- Are the illustrative skills accurate and important for the undergraduate level?
- Do illustrative skills support the competency?

**Competency 1: Ability to apply the process of science**

At the end of an immunology course, students will be able to:

IS: Locate peer-reviewed articles within the immunological sciences

IS: Distinguish between primary and secondary immunology literature

IS: Critically analyze the key findings and experimental options for diseases such as cancer, allergies, design within primary immunology literature

IS: Design an experiment to address an immunology-based research problem

**Competency 2: Ability to understand the relationship between science and society**

At the end of an immunology course, students will be able to:

IS: With evidence, debate ethical concerns of performing immunological research

IS: With data, demonstrate the impact of immunological research on society

**Competency 3: Ability to communicate and collaborate with others**

At the end of an immunology course, students will be able to:

IS: Communicate immunology in both written and oral formats at a level appropriate to the intended audience

IS: Work within a team to promote successful completion of a task related to immunology

IS: Demonstrate cooperation when working with diverse people

**Competency 5a: Ability to perform basic laboratory procedures**

At the end of an immunology course, students will be able to:

IS: Use best safety and technical practices in an immunological laboratory

IS: Use best record-keeping practices in an immunological laboratory

**Competency 5b: Ability to perform laboratory experimentation to address an immunology based research question**

IS: Isolate and identify immune cells

IS: Measure effector functions of immune components

IS: Use antigen-antibody interactions to detect the presence of an antigen or an antibody

IS: Use methodologies to manipulate the immune response

IS: Use modeling/simulation for an immunology based investigation
