# Supplementary material for: The ImmunoSkills Guide: Competencies for undergraduate immunology curricula
Source: PLoS One. 2024 Nov 11;19(11):e0313339. doi: 10.1371/journal.pone.0313339 (PMC11554037; doi:10.1371/journal.pone.0313339)
Supplement: S2 File — (DOCX) [file pone.0313339.s002.docx]

**Supporting Information**

**S2 File. Appendix 2 – Survey Instrument**

The following sections give a snapshot of the survey organization:

1. **A primer that describes the organization of the competency framework**

The organization of this hierarchical framework is depicted in Figure 1. Our task force identified six key competencies: 1. Ability to apply the process of science; 2. Ability to use quantitative reasoning; 3. Ability to communicate and collaborate with others; 4. Ability to understand the relationship between science and society; 5. Ability to perform basic laboratory procedures; 6. Ability to perform and/or explain laboratory methodology to address an immunology-based research question. For each core competency, we identified several immunology specific illustrative skills and example learning activities. These competencies may be adopted partially, or in entirety, in a course with or without a lab, depending on instructor’s class setting, learning goals (lower or upper Bloom’s level), target audience (non-majors or biology majors) or instructional time available. The following section will request your feedback on these competencies and skills.

**B) Competency 1: Ability to apply the process of science**

Q1. Please answer the following question about this competency "Ability to apply the process of science": How important is this competency for an immunology student to develop? (Not at all, slightly, moderately, very, extremely)

Q2. In your opinion, how well does the given illustrative skill align with the core competency “Ability to apply the process of science”.

i) Locate peer-reviewed articles pertaining to immunology (Not well at all, Slightly well, Moderately well, Very well, Extremely well).

ii) Distinguish between primary and secondary immunology literature (Not well at all, Slightly well, Moderately well, Very well, Extremely well)

iii) Critically analyze the key findings and experimental design within primary immunology literature (Not well at all, Slightly well, Moderately well, Very well, Extremely well)

iv) Design an experiment to address an immunology-based research problem (Not well at all, Slightly well, Moderately well, Very well, Extremely well)

Q3. Please use this box to note any other comments pertaining to the competencies and illustrative skills listed above.

**Competency 2: Ability to use quantitative reasoning**

Q1. Please answer the following question about this competency "Ability to use quantitative reasoning": How important is this competency for an immunology student to develop? (Not at all, slightly, moderately, very, extremely)

Q2. In your opinion, how well does the given illustrative skill align with the core competency “Ability to use quantitative reasoning”.

i) Apply statistics to analyze immunological data (Not well at all, Slightly well, Moderately well, Very well, Extremely well).

ii) Interpret different types of graphical representations of immunological data (Not well at all, Slightly well, Moderately well, Very well, Extremely well)

iii) Draw meaningful conclusions from an immunology-related data set (Not well at all, Slightly well, Moderately well, Very well, Extremely well)

Q3. Please use this box to note any other comments pertaining to the competencies and illustrative skills listed above.

**Competency 3: Ability to communicate and collaborate with others**

Q1. Please answer the following question about this competency "Ability to communicate and collaborate with others": How important is this competency for an immunology student to develop? (Not at all, slightly, moderately, very, extremely)

Q2. In your opinion, how well does the given illustrative skill align with the core competency “Ability to communicate and collaborate with others”.

i) Present an immunological topic to an audience (Not well at all, Slightly well, Moderately well, Very well, Extremely well).

ii) Contribute within an team to move a task forward (Not well at all, Slightly well, Moderately well, Very well, Extremely well)

iii) Contribute within a team to promote a positive environment (Not well at all, Slightly well, Moderately well, Very well, Extremely well)

iv) Demonstrate an ability to manage conflict (Not well at all, Slightly well, Moderately well, Very well, Extremely well)

Q3. Please use this box to note any other comments pertaining to the competencies and illustrative skills listed above.

**Competency 4: Ability to understand the relationship between science and society**

Q1. Please answer the following question about this competency "Ability to understand the relationship between science and society": How important is this competency for an immunology student to develop? (Not at all, slightly, moderately, very, extremely)

Q2. In your opinion, how well does the given illustrative skill align with the core competency “Ability to understand the relationship between science and society”.

i) Identify inaccuracies in popular media about immunological topics that are consumed and shared by the lay public (Not well at all, Slightly well, Moderately well, Very well, Extremely well).

ii) Discuss the impact of immunological research on society (Not well at all, Slightly well, Moderately well, Very well, Extremely well)

Q3. Please use this box to note any other comments pertaining to the competencies and illustrative skills listed above.

**Competency 5: Ability to perform basic laboratory procedures**

Q1. Please answer the following question about this competency "Ability to perform basic laboratory procedures": How important is this competency for an immunology student to develop? (Not at all, slightly, moderately, very, extremely)

Q2. In your opinion, how well does the given illustrative skill align with the core competency “Ability to perform basic laboratory procedures”.

i) Use standardized safety practices in an immunological laboratory (Not well at all, Slightly well, Moderately well, Very well, Extremely well).

ii) Use standardized technical practices in an immunological laboratory (Not well at all, Slightly well, Moderately well, Very well, Extremely well)

iii) Use standardized record-keeping practices in an immunological laboratory (Not well at all, Slightly well, Moderately well, Very well, Extremely well)

Q3. Please use this box to note any other comments pertaining to the competencies and illustrative skills listed above.

**Competency 6: Ability to perform and/or explain laboratory methodology to address an immunology-based research question**

Q1. Please answer the following question about this competency "Ability to perform and/or explain laboratory methodology to address an immunology-based research question": How important is this competency for an immunology student to develop? (Not at all, slightly, moderately, very, extremely)

Q2. In your opinion, how well does the given illustrative skill align with the core competency “Ability to perform and/or explain laboratory methodology to address an immunology-based research question”.

i) Identify and/or isolate immune cells (Not well at all, Slightly well, Moderately well, Very well, Extremely well).

ii) Measure effector functions of immune components (Not well at all, Slightly well, Moderately well, Very well, Extremely well)

iii) Detect the presence of an antigen or an antibody (Not well at all, Slightly well, Moderately well, Very well, Extremely well)

iv) Measure the immune response upon manipulation of an experimental system (Not well at all, Slightly well, Moderately well, Very well, Extremely well)

v) Use modeling/simulation for an immunology-based investigation (Not well at all, Slightly well, Moderately well, Very well, Extremely well)

Q3. Please use this box to note any other comments pertaining to the competencies and illustrative skills listed above.

**C) Demographic Questions:**

1. What is your current job title? (Retired/Emeritus, Full-time tenured faculty, Full-time tenure track faculty, Full-time non-tenure track faculty (Professor of practice, instructor, lecturer, teaching professor, etc.), Part-time faculty (adjunct, visiting professor, etc.), Post-doctoral trainee, Graduate trainee, Lab manager/technician/specialist)
2. Are you currently teaching immunology or have taught immunology in the past? (Yes, I am teaching/have taught immunology in the academic year 2020-2021; No, I have taught immunology in the past academic years; No, I plan to teach immunology in the future). And, list the courses that you are currently teaching.
3. What type of institutions have you taught at? Select all that applies. (Doctorate granting university, Master’s and Bachelor’s granting university, Primarily Bachelor’s granting university, Mixed Baccalaureate/Associate’s College, Primarily an associate’s college). Alternatively, name the institution that you are affiliated with.

**D) Please use this box to note any other comments/suggestions for our task force**
