# Supplementary material for: The ImmunoSkills Guide: Competencies for undergraduate immunology curricula
Source: PLoS One. 2024 Nov 11;19(11):e0313339. doi: 10.1371/journal.pone.0313339 (PMC11554037; doi:10.1371/journal.pone.0313339)
Supplement: S3 File — (DOCX) [file pone.0313339.s003.docx]

**Supporting Information**

**S3 File. Appendix 3** **– Interview Script**

Faculty who consented to be interviewed were invited to take the above-noted survey (Appendix 2) via Qualtrics, so they could familiarize themselves with the document. During the interview, verbal consent was gathered from each participant at the beginning of the session. The competency document was projected on the shared screen and the interviewer read out each competency and illustrative skill. The question prompts for each competency were:

1. Are the competencies and skills listed clear, scientifically accurate and important?
2. Are the illustrative skills well aligned with the core competency?
3. Do you have any comments on any of the listed competencies or skills?

The interviewer stopped and discussed the competency and/or illustrative skill if there was any pause or comment on a competency or illustrative skill, and moved on to the next one only if everything listed on the document and projected on the screen looked fine to the interviewee.
