# Supplementary material for: The ImmunoSkills Guide: Competencies for undergraduate immunology curricula
Source: PLoS One. 2024 Nov 11;19(11):e0313339. doi: 10.1371/journal.pone.0313339 (PMC11554037; doi:10.1371/journal.pone.0313339)
Supplement: S1 Table — (DOCX) [file pone.0313339.s004.docx]

**Supporting Information**

**S1 Table:** **The iteratively developed codebook used to analyze the qualitative data**

| **Code Abbreviation** | **Code Name** | **Code Description** |
| --- | --- | --- |
| MC | Missing Content | Words or phrases are missing from the core competency/illustrative skill |
| NC | Not Clear | The core competency/illustrative skill does not clearly convey the thought process or intent behind it, or the word choice is vague |
| NA | Not Aligned | The illustrative skill is not well aligned with the core competency |
| NI | Not Important | The core competency/illustrative skill is not important for undergraduate immunology education |
| OK | Consent | No issues with the core competency/illustrative skills as stated |
| NS | Not Suitable | The core competency/illustrative skill is not suitable to their course, either because of the course level or structure. |
| LA | Limitations and Accessibility | The core competency/illustrative skill cannot be covered in their course because they lack resources and/or time. |
| ND | Not Discussed | Participants did not specifically comment on this core competency/illustrative skill |
