# Supplementary material for: The ImmunoSkills Guide: Competencies for undergraduate immunology curricula
Source: PLoS One. 2024 Nov 11;19(11):e0313339. doi: 10.1371/journal.pone.0313339 (PMC11554037; doi:10.1371/journal.pone.0313339)
Supplement: S2 Table — (DOCX) [file pone.0313339.s005.docx]

**Supporting Information**

**S2 Table: Thematic analysis of focus group discussion**

| **Theme** | **Competency** | **Example Quotes** |
| --- | --- | --- |
| **Missing Content** | **1. The ability to apply the process of science** | - “Locating is easy, Google is awesome. But to vet a research article or primary or secondary literature is important” - “There is one scaffolding piece that’s missing here: once they can analyze information in one research paper and maybe one experiment within a research paper, but what they aren’t able to do is determine what the next question would be” - “So maybe there needs to be a step in the scaffold of once you find and vet an article, how then you approach it” - “One of the things that I would love to see in here, is a commentary or a statement about identifying controls, identifying the tool being used, determine whether or not it is an appropriate tool for the question being asked. If there is not a lab component, then knock it back a step to a virtual version that also lets us get at fundamental issues– why would you do a sandwich ELISA versus a capture ELISA?” |
|  | **2. The ability to understand the relationship between science and society** | - “What about, when it comes to the relationship between science and society: locate and vet reliable information for the non-immunologists?” - “Can I suggest also an additional IC that might actually be relevant to a laboratory course – such as discussing funding and what the cost of the research are? If you are teaching the students about flow cytometry, talk about the cost of antibodies, of the cytometers that are needed and at what point the cost-benefit is addressed. The cost of a biologic which is developed for immunotherapy versus the cost of traditional chemotherapy” |
|  | **5. The ability to perform basic laboratory procedures** | - “That could always be something that may be this group creates - what would be the best safety practices” - “And how about reference either to something that particular instructor has already built in before or something that they are going to be getting in a handout or a link that they could refer to and know what they are expected to do.” |
|  | **6. The ability to explain and/or perform laboratory methodology to address an immunology-based research question** | - “Adding some element of communication to this addressing immunology-based research question might be a good idea to add.” - “Each of these subpoints I see them as different experiments and each one would justify its own time devoted to it on how to write a hypothesis based on some observations over this phenomena. I don’t know if this is implied or expected but that would be something that should be emphasized whether the students are majors or non-majors.” - “Each of these points would also lend themselves really well to examples and lab exercises/experiments using invertebrates. There are a lot of really great models. When I go over immunology syllabi I see that little to any attention has been given to invertebrates” - “One thing that I don’t see here is ability to parse primary literature.” |
| **Clarity** | **1. The ability to apply the process of science** | - “I would argue that the first illustrative skill is way over complicated and detail-oriented compared to the other three. If your language is parallel, then you automatically kind of make those illustrative skills more equal.” - “Primary and secondary fall out of analyzing the literature - being able to vet it” |
|  | **2. The ability to understand the relationship between science and society** | - “The thing that I am finding challenging with immunology laboratory course is that I can see this more with an upper-division lecture class because depending on the concepts that are covered in that immunology laboratory class, they may not even be able to say that they are having data to explain the impact of immunological research in society”. - “It’s not clear why it would need to be a laboratory course to teach this core competency. You don’t necessarily have to do laboratory research to understand the relationship between science and society”. - “I don’t know if there is specific ethics from immunological research that is different from other biology fields. “When I think of ethics, there can be evidence but there is also – I don’t want someone to put a vaccine in me. Evidence is a little vague because evidence could be an article that an anti-vax group wrote. This is one of the most touchy one of any of these- the ethical stuff.” - “I am not so sure about with evidence. With evidence, it gets a little bit confusing to me. We are in a science class – do we need to specify with evidence? I would say scientific evidence would help” |
|  | **3. The ability to communicate and collaborate with others** | - “I would probably modify immunology in the first statement to say immunological principles or results or something because immunology is pretty broad” - “The third one – my question is - how do you assess that and have learning outcomes other than an argument that breakout between people” - “Diverse people is not something that you can actually set up in your course. You have who is taking your course. They may all be white males. Diverse is not something you can dictate. Diversity could be of interests”. - “Isn’t the last one sort of incorporated in the second one? In order to work with a team to promote successful completion, you need to cooperate.” - “Diversity obviously captures enormous breath of things including different perspectives, backgrounds whatever. I imagine most people reading it won’t think first of ethnic diversity, gender diversity. So, I guess the question is if that is what you are trying to capture or what is the intent of those statements.” - “Could I suggest a different order that the diversity comes first, working within a team second and communicating third” - “What do you mean by others? About the first one – what do you mean by intended audience, because there are so many different kinds of audience. Are we talking about the public, or other classmates or other professionals”? |
|  | **5. The ability to perform basic lab procedures** | - The word ‘best’ is probably not very clear. It mayIf may be ‘good’ or ‘proper.’” |
|  | **6. Ability to perform laboratory experimentation to address an immunology based research question** | - “For the fourth one - The word manipulate – I don’t know exactly what laboratory experiments you are talking about, if you are having immune cells and you are adding things to them you are manipulating. If you are looking at the immune response that has happened and you are not actually doing anything to cause change. We prefer interrogate over manipulate just because we get samples and we look to see what’s happening.” - “Would this assume that the undergraduate course actually has a lab associated with it?” - “Being able to explain is more important. Doing it is not really as important to me than understanding what’s happening.” |
| **Appropriateness** | **1. The ability to apply the process of science** | - “I am also not sure it’s really critical for an introductory immunology course at the undergraduate level. That may be something that’s more of a detail for a graduate student.” - “In a more paper-based colloquial course where I am going to have students reading a lot of papers, they are going to learn a lot about the steps of the process of science. But in a more general active learning with lecture type course especially with no lab, applying that process of science would look very different in terms of steps that I would lay out for them.” “It needs some directive for easy usability and adoption for this type of course versus that type of course, and also institutions. A lot of institutions don’t have labs that go with it.” “Design an experiment, at what level are you thinking” - “It depends entirely on the level of the course, and also the majors. I have a very much lower set of objectives for my nursing and allied health majors, because of what they need to learn coming out of immunology is fundamentally different from my bio majors who are looking at it very often in terms of pre-med school or pre-research career. I would actually love to see this one broken out a little bit more – for a 200-level course, for a 300/400 level course, something for majors, something for non-bio majors just to allow for a little more refinement and a little more focus.” |
|  | **2. The ability to understand the relationship between science and society** | - “Most people who are teaching immunology are not comfortable with teaching or dealing with ethical concerns, and to ask students to debate that is a very specific ask. So, I would argue that students should be able to identify ethical concerns related to immunological research”. “I agree that debate is an important pedagogical tool. The way I think about these kinds of exercises is not to limit or prescribe what the teacher would do to achieve that learning outcome.” - “Identifying rather than demonstrating the impact. Students don’t have that kind of intellectual reach that they wouldn’t be able to see what research might do to society unless it has already been done. That’s a very big ask for an undergrad.” |
|  | **6. The ability to explain and/or perform laboratory methodology to address an immunology based research problem** | - “These are all for majors-level course. Because for the non-majors class, or the outreach work I do – the first one is where we stop and may be a little bit of second one.” |
| **Limitations or Accessibility** | **1. The ability to apply the process of science** | - “Distinguishing primary versus secondary immunology literature; that’s absolutely something that I would consider for my Spring course because we do have them do a little bit of work….To be able to do that in a robust, in a real way, more than a few minutes you would need some sort of follow-up assignment or something, that we don’t have may be the capacity to do” |
|  | **6. The ability to explain and/or perform laboratory methodology to address an immunology based research problem** | - “Is there any opportunity to include more examples, because not every institution may be able to deal with mice, even though that’s the official immunology organism? But be able to include staining, a blood draw or flow cytometry, which also not everybody may be able to use. But just provide more examples, so somebody that may be at a small school with limited means wouldn’t have to think a ton on their own necessarily?” - “Some universities may not have the resources or the capacity to have even vertebrate animals even for a short amount of time. So maybe the possibility of some comparative immunology where you use other models that may not necessarily be the most common ones, like snails that might be easier to keep in a fish tank or something. And being invertebrate you don’t need a lot of permits.” - “With the cost of reagents and the cost of some of the equipment in a four-year liberal arts school, where many of these students aren’t going to go on in research, so to understand how to do it but actually physically do it is another matter” - “For the fourth point: Even injecting antigens into mice is beyond what we can do in a teaching lab. So if that’s what they are talking about – financially and in terms of facilities – we can’t afford to do that.” |
| **Consent** | **2. The ability to understand the relationship between science and society** | - “I like it. There are very few students who are enthusiastic about working with data. They can connect it to real life. So, this is really cool.” |
|  | **3. The ability to communicate and collaborate with others** | - “This is a great competency fundamentally.” |
|  | **6. The ability to perform laboratory methodology to address an immunology-based research question** | - “There is some pretty cool stuff out there related to epidemiology and infection rates that I think modeling and simulation would be really good. I like seeing that included because I think that’s going to be incredibly important moving forward from this pandemic situation.” |
