# Supplementary material for: The ImmunoSkills Guide: Competencies for undergraduate immunology curricula
Source: PLoS One. 2024 Nov 11;19(11):e0313339. doi: 10.1371/journal.pone.0313339 (PMC11554037; doi:10.1371/journal.pone.0313339)
Supplement: S3 Table — (DOCX) [file pone.0313339.s006.docx]

**Supporting Information**

**S3 Table: Thematic analysis of interview data**

| **Theme** | **Competency** | **Example Quote** |
| --- | --- | --- |
| **Missing content** | **1. The ability to apply the process of science** | - “I like the idea of explicitly asking students to think about how implicit bias and the structural and institutional hierarchies in which science is done in, shapes how we interpret the results and design experiments. For example, the baseline for spirometry is discounted by 10% for individuals of African descent and the argument is rooted in the idea of something fundamentally different because of genetics, but it is actually environmentally driven - the way environmental justice works out in our society, where air pollution exists, exposure to early toxicants, and our society is spatially structured around race.” |
|  | **2. The ability to understand the relationship between science and society** | - “This is the one where I really think the implicit bias pops out. The victim blaming we use for some diseases and the non-victim blaming language we use for others and how that affects our interpretation of results, and our funding and how the public perceives it. I like the idea of making it explicit.” |
|  | **4. The ability to use quantitative reasoning** | - “Yes, these are good, in light of our previous conversation about implicit bias.” |
|  | **6. The ability to apply explain and/or perform laboratory methodology to address an immunology-based research question** | - “Every semester I always make sure I add this is histology. Immune education has to have a histological component to it” |
| **Clarity** | **1: The ability to apply the process of science** | - “Only thing that I paused for a bit was the term secondary. I never heard that before. I have used primary before. I guess whatever is not primary is secondary” |
|  | **3. The ability to communicate and collaborate with others** | - “Present an immunological topic to an audience – I’d like to explicitly also say that not just to a scientific audience but also to a public audience.” - “I am thinking about 3.3 – how would you measure the promotion of a positive environment” - “Well that one is interesting – an ability to manage conflict – what’s that about” |
|  | **6. The ability to perform and/or explain laboratory methodology to address an immunology-based research question** | - “I think by writing this competency as ability to perform and/or explain – those are two very separate things for me. I think the ability to explain laboratory methodology is a core competency for undergraduate immunology students. Everything that is listed here – except for modeling – an undergraduate student in immunology should be able to do it. But perform – I find that to be a very high bar.” |
| **Appropriateness** | **3. The ability to communicate and collaborate with others** | - “For non-majors – I have them do group work in class, so they are definitely collaborating. But the examples are too high level for the class that I was thinking of’’ |
|  | 1. **The ability**   **to use quantitative reasoning** | - “I teach the non-majors the most – that’s where my mindset is coming from. I do have them look at some histograms and stuff, but its super low-stakes – just introducing the concept of it”. - For 4.1: “So, we are going into more biostatistics aspect of it. I don’t think my undergraduate students will be using that as much in the particular course that I am teaching.” - “The 4.1 is sometimes beyond what I would teach in an immunology class to undergraduates. I would like to teach that to them but I don’t always.” |
|  | 1. **The ability**   **to perform basic lab procedures** | - “I teach the non-majors the most – that’s where my mindset is coming from. They’ll get it somewhere else, maybe not Immuno-related.” - **“**I do have some thoughts about whether immunology labs are essential for undergraduate immunology courses, simply because I know many immunology courses don’t teach labs. I don’t know that I agree with Vision and Change that this competency is a competency for all undergraduate immunology students.” |
|  | 1. **The ability**   **to explain and/or perform laboratory methodology to address an immunology based research problem** | - “So even when we were teaching immunology labs in the past I could clearly see top 4 being implemented there. But I don’t think 6.5 would be easily used in teaching a lab. But at the same time I have to emphasize that it is absolutely important to have something like simulation for students, because I think that is more effective. Just like primer design, I wish we had something for immunology too, where they could see different responses.” |
| **Limitations or Accessibility** | **6. The ability**  **to explain and/or perform laboratory methodology to address an immunology based research problem** | - “While all of the illustrative concepts are very solid, many institutions will not be able to support this type of laboratory work. If you can find people who teach immunology now who had to go virtual, and partner with them and find units that check each of these boxes, that would be great marketing for them because they are out there” - “You can still understand what’s going on, I think, without the hands-on experience. It’s not fun. There have been some decent virtual lab experiences. I have seen some ELISA stuff, and some blood typing and flow. You can still think about all that stuff and understand it, which ultimately is more important” - “We work very hard to bring in data and applications, and materials for ELISA, so students can see them and have that connection. But, we have never been allowed – they told us not in the budget – for the lab” |
| **Consent** | 1. **The ability to apply the process of science** | - “There are going to be people who teach immunology with no lab. So, for them finding literature and all that is really important and should be emphasized.” |
|  | 1. **The ability to understand the relationship between science and society** | - “Yeah. Those are fantastic” |
|  | 1. **The ability to communicate and collaborate with others** | - “I like that. Those are good” |
|  | 1. **The ability to use quantitative reasoning** | - “Looks good” |
|  | 1. **The ability to perform basic lab procedures** | - “I think those are all really valuable” |
|  | 1. **The ability to explain and/or perform laboratory methodology to address an immunology-based research question** | - “I really like how these are written, and that they could be done with or without the lab, except for technical manipulation.” |
